# Comparison of Ocular and Brain Abnormalities Among Neonates With In Utero Exposure to Opium or Other Drugs

**DOI:** 10.7759/cureus.27648

**Published:** 2022-08-03

**Authors:** Maryam Khoshnood-Shariaati, Sahar Ashrafzadeh, Afsar Dastjani-Farahani, Robabe Zamani, Ali Naseh

**Affiliations:** 1 Pediatrics, Neonatology, Shahid Beheshti University of Medical Sciences, Tehran, IRN; 2 Department of Psychiatry, David Geffen School of Medicine, University of California, Los Angeles, USA; 3 Ophthalmology, Farabi Eye Center, Tehran University of Medical Sciences, Tehran, IRN; 4 Pediatric Medicine, Shahid Beheshti University of Medical Sciences, Tehran, IRN; 5 Pediatrics, Mofid Children Hospital, Shahid-Beheshti University of Medical Sciences, Tehran, IRN; 6 Pediatrics, Taleghani Hospital, Shahid-Beheshti University of Medical Sciences, Tehran, IRN

**Keywords:** in utero drug exposure, neonate, pregnancy, addiction, brain sonography, ocular abnormalities

## Abstract

Background

There are growing concerns regarding ocular and brain abnormalities in infants who had in utero exposure to various kinds of substances/drugs. We compared the ocular and brain abnormalities among neonates based on the type of drug used by mothers.

Methodology

This prospective cohort study of 305 neonates included all neonates at Mahdieh Hospital, Tehran, Iran, who had their records for ophthalmic screening and brain sonography and were born to mothers with a history of substance use disorder (2014-2017). Demographic data, results for viral antibodies (human immunodeficiency virus, hepatitis C, and hepatitis B), and Apgar scores at one and five minutes were collected. We excluded neonates with Apgar score <8 at one minute, weight <1,800 g, gestational age <35 weeks, asphyxia, or anomalies. The neonates’ eyes were examined using tropicamide 0.5%, phenylephrine 2.5%, and tetracaine.

Results

The prevalence of substance use disorder among pregnant women was 1.8%. The study included 305 neonates with a mean gestational age of 37.8 ± 1.6 weeks, while the mean age of their mothers with substance use disorder was 29.8 ± 6.4 years. Ophthalmologic examination showed that 37 (12%) neonates had abnormal incomplete retina vascularization, and brain abnormalities were seen in 29 (9.5%) neonates; however, no difference was identified based on the type of drug used by mothers. The birth weight (BW) of the neonates depended on the type of drugs used by the mothers (p = 0.027). Maternal use of cannabis and amphetamine were associated with the lowest and highest BWs (2,800 ± 283 and 3,750 ± 42 g), respectively.

Conclusions

The BW of neonates depended on the type of drugs used by the mothers, where cannabis and amphetamine use were associated with the lowest and highest BWs, respectively. However, our data could not identify if neonates’ ocular and brain abnormalities differed based on the types of drugs. This study highlights the importance of a drug-free pregnancy and the need for addiction-prevention programs provided to women of childbearing age.

## Introduction

Substance use disorder during pregnancy is a continuation of a chronic recurrent disease characterized by features including the compulsion to seek and abuse drugs, loss of control over restricted use, and the development of unpleasant behaviors such as anxiety and irritability [1].

According to various studies, the prevalence of substance use disorder among women, most of whom are of childbearing age, has increased significantly in the last two decades [2]. Therefore, women are an important group to be considered while planning treatment programs for individuals with substance use disorder. However, in most countries, addiction treatment and harm reduction programs tailored to the needs of women with substance use disorder are either non-existent or very rare. Pregnancy in women with substance use disorder is much riskier compared to women without substance use disorder. If a mother goes through pregnancy without giving up drugs, and then those drugs do not cause her to give birth to a stillborn child, her surviving child will definitely have physical and mental problems that may develop at later stages of the child’s life [3].

Opioid addiction is one of the health and social problems of the current century and can affect the physical and mental aspects of individuals. When addiction occurs among pregnant women, the health of their babies may become affected too. Substance use disorder imposes significant costs on society, and each year billions of dollars are spent on treating drug addicts and addressing the consequences of substance abuse [4,5].

Opioid-addicted women face more health issues compared to non-addict women during their childbearing age and pregnancy, including problems with ovulation, menstrual irregularities, and early menopause [6]. Moreover, the effects of substance use disorder in women are more important than in men because of the possibility of pregnancy and the risks that may affect their fetuses, including causing low birth weight, preterm delivery, malnutrition, miscarriage, or withdrawal syndrome [7].

Previous studies have shown a significant association between methadone addiction in mothers and visual disturbances in their neonates. Studies have reported that nystagmus and strabismus are common ocular abnormalities among these neonates [8,9]. An epidemiological study reported that the prevalence of ophthalmic complications among healthy neonates in the Middle East is around 2.14% [10]. Examples of those abnormalities include intraretinal hemorrhages, nasolacrimal duct obstruction, refractive errors, congenital cataracts, or ptosis. Additionally, the prevalence of brain abnormalities such as craniosynostosis among neonates has been reported to be around 0.15% [11]. This study aimed to compare ophthalmology and brain findings and their prevalence in neonates who had intrauterine exposure to opium and other drugs.

## Materials and methods

This prospective cohort study included all neonates born to mothers with substance use disorder in Mahdieh Hospital affiliated with Shahid-Beheshti University in Tehran, Iran, from 2014 to 2017. The study included 305 neonates. Substance use disorder was determined based on the medical history of the mothers and the information documented in the neonate’s file.

We excluded neonates with gestational age <35 weeks, birth weight <1,800 g, Apgar score <8 in the first minute after delivery, those born to mothers who used group D drugs during pregnancy (which include prescription drugs needed to treat serious diseases in mothers), those with missing information in patients’ records, those born to mothers addicted to cocaine (in Iran, cocaine is an uncommon drug), those who experienced asphyxia, or those who had other anomalies.

Demographic data such as gestational age, sex, birth weight, mean Apgar score at the first and fifth minutes, human immunodeficiency virus (HIV) antibody, hepatitis C antibody, hepatitis B antibody, brain sonography, ophthalmic examination of ocular abnormalities including abnormal incomplete vascularization of the retina, maternal age, and history of maternal substance use disorder (type of substance and duration of usage) were collected. Examination of the eyes of neonates was performed through indirect ophthalmoscopy by opening the pupil with a combination of tropicamide 0.5% drop, phenylephrine 2.5% drop, and tetracaine drop.

Data analysis

Results are presented as mean for numeric variables, or absolute frequencies and percentages for categorical variables. Numeric variables were compared using the independent two-sample t-test. Categorical variables were compared using a chi-square test across binary groups or a chi-square test for trends across ordinal groups. For the statistical analysis, the statistical software SPSS version 25.0 (IBM Corp., Armonk, NY, USA) was used. All p-values were considered two-tailed, with a statistical significance level set at 0.05.

This study was approved by the Ethics Committee of Shahid-Beheshti University of Medical Sciences (IR.SBMU.MSP.REC.1398.719).

## Results

The prevalence of substance use disorder among pregnant women was 1.8%. In total, 305 neonates met the inclusion criteria and were included in the study. The mean gestational age of the neonates was 37.8 ± 1.6 weeks, of whom 141 (46%) were females, and 164 (54%) were males. The mean birth weight of neonates was 2,916 ± 474 g. The mean first-minute Apgar score was 9 ± 0 with a range of 8-10. There were 305 addicted mothers with a mean age of 29.8 ± 6.4 years. In total, 123 (40%) had a cesarean section delivery, and 182 (60%) had a vaginal delivery.

Table 1 presents the type of substances that mothers used, where opium was used by 23% of mothers. Table 2 compares the averages for gestational age, birth weight, and Apgar score based on the type of drug the mother used.

**Table 1:** Types of substances used by mothers (n = 305).

Substance	N (%)
Opium	62 (23%)
Multiple drugs	134 (50%)
Heroin	15 (6%)
Alcohol	11 (4%)
Cannabis	2 (0.7%)
Amphetamine	3 (1%)
Methamphetamine	14 (15%)

**Table 2:** Association between the type of drug used by mothers and neonatal characteristics (n = 305). *P-values less than 5% are considered significant. Neonates whose mothers used cannabis had the lowest and neonates whose mothers used amphetamine had the highest birth weights.

	Alcohol (n = 11)	Methamphetamine (n = 14)	Opium (n = 62)	Heroin (n = 15)	Cannabis (n = 2)	Multiple drugs (n = 134)	Amphetamine (n = 3)	P-value
Gestational age (weeks)	38.36 ±v 1.21	37.94 ± 1.37	38.02 ± 1.55	37.13 ± 1.89	37.50 ± 0.71	37.55 ± 1.74	37.67 ± 0.58	0.332
Birth weight (g)	2,992 ± 667	3,093 ± 494	2,873 ± 447	2,917 ± 443	2,800 ± 283	2,866 ± 451	3,750 ± 42	0.027*
Apgar score	9 ± 0	9 ± 0	9 ± 0	9 ± 0	10 ± 1	9 ± 0	9 ± 1	0.312

The mean gestational age of neonates showed that mothers addicted to alcohol and heroin had the highest and lowest gestational ages, respectively, although this difference did not reach statistical significance (Table 2).

The mean birth weight of neonates based on the type of drugs their mothers used showed a statistically significant difference between these groups (p = 0.027). The lowest and highest of these averages were related to maternal use of cannabis (marijuana) and amphetamine, respectively (Table 2).

Ocular abnormalities

In the eye examination, 37 (12%) had posterior segment abnormalities, such as abnormal retinal revascularization. Of this, 30 neonates who had incomplete vascularization in zone three had a gestational age of 38-40 weeks, five neonates who had incomplete vascularization in zone two had a gestational age of 36-38 weeks, and two neonates who had incomplete vascularization in zone one had gestational ages of 35 and 36 weeks. All 30 neonates who had incomplete vascularization in zone three reached complete vascularization during follow-up visits. The remaining 268 (88%) neonates had no anterior or posterior segment ocular abnormalities. This study could not identify any association between the type of substance used by mothers and the presence of ocular abnormalities in neonates (p = 0.828).

Brain abnormalities

The results of brain sonography showed that 29 (9.5%) neonates had brain abnormalities, including 13 (4.3%) neonates who had an intraventricular hemorrhage, two (0.7%) had hydrocephaly, 11 (3.6%) had brain cysts, one (0.3%) had brain calcification, and two (0.7%) had periventricular leukomalacia. Moreover, this study could not identify any association between neonatal brain abnormalities and the type of drug used by their mothers who had substance use disorder (p = 0.523).

Maternal viral biomarkers

Evaluation of mothers for the presence of viral biomarkers showed that three (1%) mothers were positive for biomarkers, including one positive for HIV who was addicted to multiple drugs, one mother was positive for hepatitis C virus who was addicted to alcohol, and one mother was positive for hepatitis B virus who was addicted to heroin. However, this study could not identify any association between viral biomarkers and the type of drug used by mothers (p = 0.259).

Additionally, this study could not identify any association between ocular abnormalities and brain abnormalities (p = 0.196) nor any association between ocular abnormalities and maternal viral biomarkers (p = 0.172). Further, the Apgar score did not show any difference between the groups.

## Discussion

This study evaluated 305 neonates who were born to mothers with substance use disorder and found a significant association between the type of drug used by the mothers and their neonates’ birth weight. The lowest and highest birth weight averages were related to the maternal use of cannabis and amphetamine, respectively. Keegan et al. reported that drug addiction increases the prevalence of low-birth-weight babies’ deliveries, which is consistent with our findings [12].

However, our data could not identify if the neonates’ ocular and brain abnormalities differed based on the type of drugs used by mothers. Our data showed that a high rate (12%) of the neonates born to mothers with substance use disorder had eye complications compared to the 2.14% prevalence, which is reported in the literature among healthy neonates [10]. Additionally, the prevalence of brain abnormalities among our neonate patients was 9.5%, which was much higher than the 0.15% prevalence reported among the neonate population [11]. The high prevalence and heterogeneous brain abnormalities present in this study are important and valuable findings that can be studied further in future research.

Ocular abnormalities such as incomplete retina vascularization were seen in 37 neonates. This study showed that 30 of those neonates who had incomplete vascularization in zone three had normal retinal vascularization during follow-up visits. We could not identify any significant difference between the type of ocular abnormalities based on the type of drug used by mothers, perhaps due to the relatively small sample size (p = 0.894). A meta-analysis by Anderson et al. [13] reported that children whose mothers were exposed to drugs experienced a higher rate of vision problems. Because we did not have a control group, we were unable to perform such an analysis.

Further, we could not find any association between brain abnormalities based on the type of drug used by mothers. A study by Tandon and Mulvihill [14] found that there was a significant association between brain and ocular abnormalities in infants who were exposed to drugs before being born. Our related data could not reach statistical significance.

Another study by Torshizi et al. [15] found that women with substance use disorder had a significantly higher prevalence of premature deliveries (p ≤ 0.01). Their study showed that the odds ratio (OR) for premature delivery was 5.96 times higher for women with substance use disorder. That study had a different design compared to ours because we excluded neonates with a gestational age of fewer than 35 weeks.

Some scientific sources have reported that there are consequences for fetal alcohol syndrome that last for a lifetime and include physical, mental, and behavioral concerns [16]. However, some other sources have defined fetal alcohol spectrum disorder that may present as a wide range of effects. Partial disorder refers to children who have only two of the physical aspects of fetal alcohol syndrome. They experience slow growth and problems with their central nervous system. The other side of the spectrum includes children who experience severe effects due to alcohol consumption by their mothers, such as fetal death, abnormalities of their face, growth problems, and central nervous system problems that cause learning and mental disabilities [17]. Our study sample included several neonates who were born to mothers who used alcohol during their pregnancy. Although not statistically significant, those neonates had the highest gestational age among all neonates in our study sample.

Three of the mothers with substance use disorder had positive viral biomarkers, which included one positive mother for each of HIV, hepatitis C virus, and hepatitis B virus. This study could not find any association between maternal viral biomarkers and the type of drug used by mothers. Perhaps this was because the number of mothers who had the biomarkers was not large enough to reach any statistical significance.

Another study reported that the effects of prenatal exposure to drugs on the developmental parameters of the offspring are drug-dependent. The study stated that different effects are due to the different pharmacological effects of different types of drugs [18]. However, our sample was not large enough to detect potential associations between specific drugs and their effects on neonates.

Some factors that may hinder the collection of accurate data in a similar prospective study setting include the fact that during a pregnancy, it is difficult for clinicians to determine for how long and for what dosage the substance has been used by the pregnant woman. In those cases, not only it is difficult to determine the type of drug use but it also needs the evaluation of other biopsychosocial factors such as socioeconomic status, educational level, biological factors, prenatal care received, and nutritional status, which also contribute to fetal and neonatal outcomes and vary from individual to individual. Hence, very little information is available about the effects of maternal substance use disorder on the fetus and neonate, considering the above confounding factors.

Study limitations

This study was a prospective single-center study and mothers had little involvement in this study. Collecting detailed information about drug use directly from mothers would have strengthened our data. Larger studies may better identify associations as a small sample cannot reach statistical significance. Moreover, the results for neonatal eye examinations were based on the initial examinations and clinical records from the time of their birth, while maternal substance use disorder during pregnancy can cause ocular complications that develop after infancy. Therefore, we suggest that future studies should evaluate the incidence of ocular complications in children born to mothers with substance use disorder upon them entering school to better show the long-term effects of drug use during pregnancy.

## Conclusions

Our study reported that the prevalence of substance use disorder among pregnant women was 1.8%. Neonates’ birth weight depended on the type of drugs their mothers used. Maternal use of cannabis and amphetamine were associated with the lowest and highest birth weights, respectively. However, our data could not identify if neonates’ ocular and brain abnormalities differed based on the type of drugs used by mothers. This study highlights the importance of a drug-free pregnancy and the need for addiction prevention programs provided to women of childbearing age.
